# Incidental Papilledema Revealing Neurotuberculosis: A Case Report of a Diagnostic Challenge

**DOI:** 10.3390/diagnostics16142199

**Published:** 2026-07-14

**Authors:** Lucia Ambrosio, Serena Panariello, Emanuela Mattiello, Luca D’Andrea, Eugenia Bruzzese, Andrea Lo Vecchio, Mario Damiano Toro

**Affiliations:** 1 Division of Ophthalmology, Department of Public Health, University of Naples Federico II, Via Sergio Pansini 5, 80131 Naples, Italy; mariodamiano.toro@unina.it; 2 Ophthalmology Unit, Department of Neurosciences, Reproductive Sciences and Odontostomatology, University of Naples Federico II, 80131 Naples, Italy; sere.panariello@gmail.com (S.P.); emanuelamatt93@hotmail.com (E.M.); 3 Department of Life Science, Health and Health Professions, Link Campus University, 00165 Rome, Italy; l.dandrea@unilink.it; 4 Department of Translational Medical Sciences, University of Naples Federico II, 80131 Naples, Italy; eugbruzz@unina.it (E.B.); andrea.lovecchio@unina.it (A.L.V.)

**Keywords:** papilledema, pediatric miliary tuberculosis, ocular ultrasound, brain MRI

## Abstract

**Background**: Tuberculosis (TB) remains a major global health problem, particularly in children, who are at higher risk of extrapulmonary and disseminated disease. Ocular tuberculosis is a rare manifestation and often presents with nonspecific signs, leading to delayed diagnosis. **Case Presentation**: We report the case of a five-year-old previously healthy boy presenting with a two-month history of persistent fever. Initial investigations revealed a positive tuberculin skin test and interferon gamma release assay, with chest CT findings suggestive of miliary TB. Despite negative microbiological tests, antitubercular therapy was initiated. Ophthalmologic evaluation showed preserved visual acuity, bilateral mild papilledema, and multifocal chorioretinitis. Neuroimaging confirmed central nervous system involvement with disseminated tubercular lesions. Fundus examination revealed grade 1 papilledema and chorioretinal lesions. Ocular ultrasound demonstrated an increased optic nerve sheath diameter consistent with intracranial hypertension. Brain MRI showed intra-axial tubercular dissemination. Extensive infectious and immunological testing excluded alternative diagnoses. The patient received prolonged multidrug antitubercular therapy, including isoniazid, rifampicin, ethambutol, pyrazinamide, amikacin, levofloxacin, and corticosteroids. Treatment was continued for a total of 15 months. Ocular findings regressed within two months, and complete systemic resolution was achieved by 21 months. At a five-year follow-up, the patient remained in good health with normal neurodevelopment and no disease recurrence. **Conclusions**: This case underscores the importance of considering ocular TB in children with prolonged fever and subtle ocular findings. Early ophthalmologic evaluation and prompt initiation of antitubercular therapy are essential to prevent severe complications and ensure favorable outcomes.

## 1. Introduction

Tuberculosis (TB) is a chronic granulomatous infection caused by *Mycobacterium tuberculosis* (MTB) that still represents a major issue in public health. It is estimated that one-third of the world’s population is infected with MTB [1]. In 2023, 10.8 million people were affected globally, with an incidence of 134 per 100,000 and a mortality rate of 11.6%. Children account for about 15% of global TB-associated deaths (191,000 deaths in 2023) [2]. In Europe, children under 15 years accounted for 4.3% of cases, with a 10% increase in pediatric TB in 2023 compared to the previous year [3].

Although pulmonary TB is the most common clinical presentation, children are at increased risk of developing extrapulmonary localization, and, mainly in infants and younger children, multiple organs can be involved through miliary dissemination. The incidence of severe neurological sequelae due to central involvement is higher in children, especially in those under five years old [4], as well as the death rate, because of the immaturity of their immune system [5,6]. Moreover, the diagnosis of pediatric TB is often challenging because of non-specific clinical manifestations, thus leading to a delay in the start of therapy and a poorer prognosis.

Ocular tuberculosis is a rare extrapulmonary manifestation. Both the anterior and posterior segments can be involved. Even the eyelids and lacrimal glands can be affected, especially in children, where diffuse infiltration simulating preseptal cellulitis, dacryoadenitis, or lacrimal gland abscess has been reported [7].

Among severe intraocular manifestations, vitritis with snowballs and snow banking, vasculitis, cystoid macular edema, and retinochoroiditis have been well described. Ocular TB is known to be a great mimicker and should be considered in the differential diagnosis of a variety of inflammatory and infectious diseases: tubercles, multifocal choroiditis, and serpiginous-like choroiditis can occur, as well as papillitis and macular scar [8]. Optic neuropathy, including edema of the optic nerve head and consequent optic atrophy, may be due to increased intracranial pressure due to tubercular meningitis, obstructive hydrocephalus, or multiple tuberculomas in the brain [9].

Recognition of ocular TB as a clinical manifestation of extrapulmonary TB is critical, as prompt detection can lead to early initiation of antituberculosis therapy (ATT) and prevent poor patient outcomes. Prompt ATT administration in ocular TB is, in fact, associated with positive outcomes and a reduction in recurrence, especially in immunocompetent patients [10]. In cases of suspected ocular TB, diagnosis is supported by a positive immunological assay for a response to MTB (i.e., tuberculin skin testing—TST and/or interferon gamma release assays—IGRA), and microbiological evidence of MTB through culture or, mainly, polymerase chain reaction (PCR), to detect mycobacterial genome from small samples of ocular fluid, even though it can still lead to false negatives. Chest X-ray is recommended to assess occult pulmonary lesions [11]. Fluorescein angiography is indicated to evaluate the presence of active choroidal lesions and retinal vascular leakage, while ultrasonography can be useful in revealing moderate to low internal eye reflectivity of large tuberculomas [12].

Although in cases of overt disease ophthalmologists are generally aware of the need to request tuberculosis testing, in more subtle or atypical presentations, the diagnosis may be overlooked. Detection of even mild disc swelling or small peripheral chorioretinal lesions may be the first sign of central involvement, thus prompting a more aggressive treatment approach. This is especially relevant in patients with unexplained systemic symptoms, such as fever, vomiting, or neurological irritability, in whom routine fundus evaluation might be deferred.

Therefore, herein we report a pediatric case of atypical ocular tuberculosis: a five-year-old child with persistent fever, asymptomatic for ocular involvement.

## 2. Case Report

A five-year-old otherwise healthy boy, HIV-negative and of Italian origin, with a two-month history of persistent fever, was referred for diagnostic purposes and presented to our ophthalmology clinic for a consultation from the pediatric department.

Two months before, the boy was hospitalized for recurrent fever in another pediatric clinic, where he tested positive for EBV serology (IgM and IgG). However, after one month of persistent fever, an extensive immuno-rheumatological and infectious disease screening was performed. Although the chest X-ray showed only mild and diffuse thickening of the bronchovascular markings, with no evidence of definite active focal lesions (initially reported as negative), the positive TST (induration diameter 15 mm) prompted referral to our institution, which hosts the Regional Referral Center for pediatric TB. Here, a diagnosis of active TB was supported by a positive IGRA (QuantiFERON-TB Gold Plus: TB1 = 10 IU/mL, TB2 > 10 IU/mL) and by a chest CT scan demonstrating reduced lung transparency due to diffuse microgranularity with ill-defined margins throughout the lung fields, along with clusters of calcifications (3 mm each) in the left mediastinal area anterolateral to the aortic arch. Positive results of the Gene-Xpert-RIF Ultra test and positive staining on multiple gastric aspirates were collected. Mycobacterium tuberculosis was not detected in cerebrospinal fluid (CSF) samples, possibly owing to the delayed performance of the lumbar puncture.

Oral ATT was started with isoniazid, rifampicin, ethambutol, and pyrazinamide. Due to the lack of clinical response and the presence of increased inflammatory markers and persistent fever, intravenous antibiotic treatment was started, first with ceftriaxone and then with ciprofloxacin. During hospitalization, other infections, including EBV, CMV, HIV, syphilis, toxoplasma, as well as bacterial infections, were ruled out.

**Figure 1:**
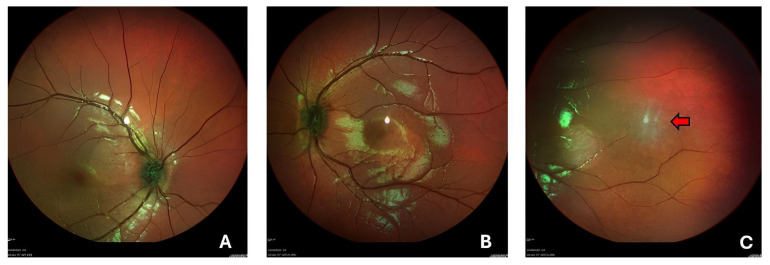
Fundus photographs. Fundus photographs were taken two weeks after diagnosis and the beginning of therapy for miliary tuberculosis. (**A**,**B**) Photographs of the posterior pole show bilateral grade 1 papilledema, with mild swelling of the optic nerve head in the superior sector. (**C**) In the temporal periphery of the left eye, two small whitish patches of chorioretinitis are visible (arrow).

**Figure 2:**
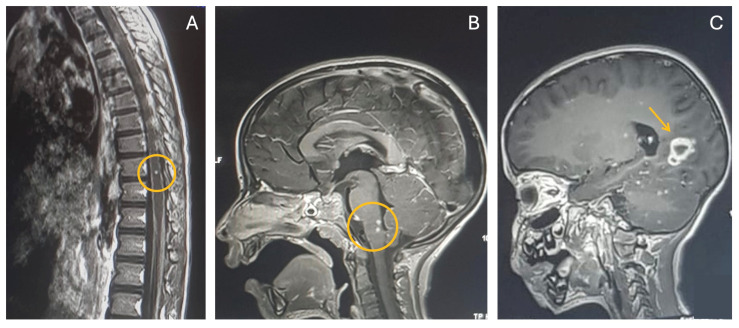
Neuroimaging features of TB with central nervous system localization performed at the beginning of anti-TB treatment. (**A**) Intramedullary tuberculous abscess of the spinal cord, appearing as a focal lesion with well-defined margins (yellow circle), consistent with inflammatory infectious involvement. (**B**) Multiple TB abscesses involving the brainstem, localized at the level of the pons (yellow circle). (**C**) Large, multilobulated tuberculous abscess involving the occipital lobe (yellow arrow), associated with contrast enhancement and mass effect on the surrounding parenchyma.

In addition to persistent fever, after ten days, the child presented with persistent irritability and occasional vomiting and mild hyponatremia, which prompted a referral for ophthalmologic evaluation. Best corrected visual acuity (BCVA) was 20/20 in both eyes; no pupillary defect or strabismus was seen; no sign of anterior chamber inflammation was detected. Fundus ophthalmoscopy showed bilateral grade 1 papilledema, with mild swelling of the optic nerve head, a C-shaped halo surrounding the disc sparing the temporal margin, and patches of multifocal chorioretinitis (Figure 1). Ocular ultrasound and brain MRI were recommended. At ultrasound evaluation, optic nerve dimensions were 4.45 mm in the right eye and 4.60 mm in the left eye (normal value 4.2 mm), confirming ongoing intracranial hypertension. MRI revealed intra-axial tubercular dissemination, involving both the brain and the spinal cord, with a main brain caseous lesion. The picture was compatible with miliary tuberculosis (Figure 2). EEG showed, while awake, sporadic slow anomalies on the left frontal and temporo-occipital regions.

Due to central nervous system involvement secondary to miliary TB, the anti-tubercular therapy was adjusted by switching to intravenous rifampicin and isoniazid, with the addition of amikacin (for up to one month), levofloxacin (forup to three months), and dexamethasone (for four weeks followed by tapering). In addition, oral treatment with pyridoxine, pyrazinamide, and ethambutol was continued. The latter was then discontinued once the susceptibility test became available.

Ten days after the introduction of specific treatment, a new ophthalmologic examination was executed, revealing a regression of papilledema, but persistent areas of chorioretinitis. Intravenous therapy was gradually transitioned to the oral route. The overall anti-tubercular treatment lasted 15 months, including an induction phase of 3 months with four drugs, followed by a 12-month continuation phase with two drugs. During follow-up, multiple clinical, biochemical checks, as well as ophthalmology consultations and MRI scans were performed.

**Figure 3:**
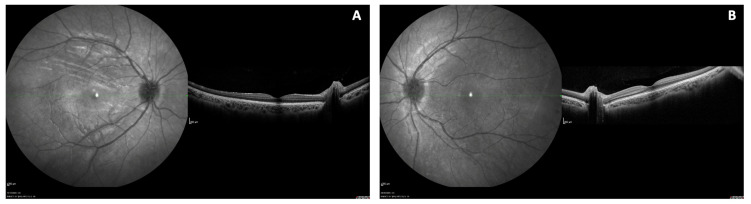
Bilateral SD-OCT B-scans of the optic nerve head showing resolving papilledema after treatment: reduced disc elevation, attenuated anterior deformation of the peripapillary Bruch’s membrane, and progressive normalization of the peripapillary contour. (**A**) Right eye; (**B**) Left eye.

A regression of the ocular involvement was achieved in 2 months (Figure 3), while complete regression of the systemic disease was observed after 21 months from diagnosis. Five years after the diagnosis, the child was in good clinical condition, showed age-appropriate neurodevelopment, and no relapses had been recorded.

## 3. Discussion

**Figure 4:**
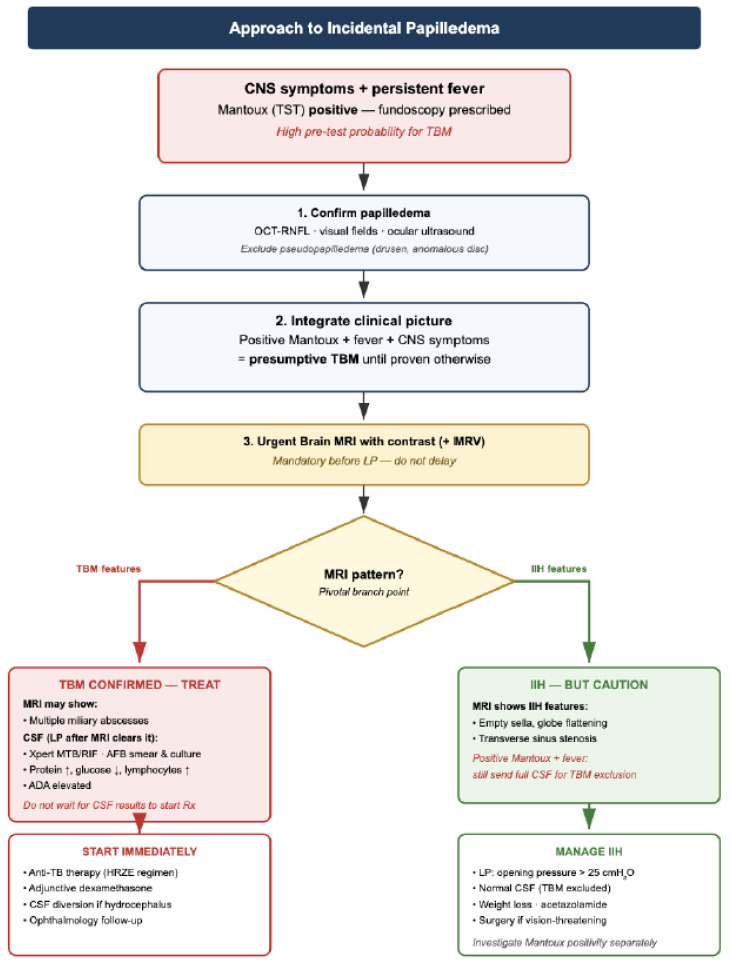
Diagnostic algorithm for incidental papilledema. After ophthalmologic confirmation and clinical reassessment, contrast-enhanced brain MRI with MRV directs management: a TBM pattern (basal enhancement, tuberculomas, hydrocephalus) prompts CSF analysis and anti-tubercular therapy with dexamethasone, whereas an IIH pattern (empty sella, transverse sinus stenosis) is confirmed by elevated opening pressure with normal CSF. This algorithm represents the authors’ proposed approach based on current evidence.

Our case outlines some of the main issues in the everyday pediatric and ophthalmological practice. First of all, ocular TB may be asymptomatic, and visual acuity may be preserved. For this reason, even mild papilledema, especially in a child with systemic symptoms, must be assessed, as it might be the first detected sign of intracranial involvement. This proves the necessity of a multidisciplinary approach, including ophthalmologists, since their evaluations may change the diagnostic pathway and treatment (Figure 4).

### 3.1. Ocular Tuberculosis: The Great Mimicker

Ocular TB is known to be a great imitator with dramatic outcomes in cases of delayed diagnosis. In 80% of cases, no pulmonary manifestation is associated [13], thus a negative chest X-ray for active disease cannot rule out the diagnosis.

Ocular manifestations include both anterior and posterior involvement: findings such as phlyctenular keratoconjunctivitis with keratolysis, serpiginous-like choroiditis [13], retinal vascular occlusion, choroidal tubercles, multifocal choroiditis, subretinal abscesses, neuroretinitis, optic neuritis, and panuveitis have been reported in pediatric patients [14,15]. Neuro-ophthalmic manifestations such as papilledema, papillitis, optic nerve tuberculoma, or optic nerve involvement secondary to intracranial hypertension or tuberculous meningitis have been described [16]. All these findings are nonspecific for TB: autoimmune and inflammatory diseases such as sarcoidosis, infectious uveitis such as toxoplasmosis and viral retinitis, idiopathic intracranial hypertension, and optic neuropathies must be ruled out. Diagnosis is essentially clinical, supported by immunological testing, systemic imaging such as chest X-ray and MRI, exclusion of differential diagnoses, and response to therapy.

The most common symptoms of ocular TB are decreased vision and ocular pain [17]. Our patient was a rare case of asymptomatic ocular involvement, with preserved visual acuity. This is significant: in everyday pediatric clinical practice, fundus examination may be deferred in patients with no symptoms or signs of ocular involvement. However, the absence of vision loss should not exclude optic neuropathy, as papilledema might be asymptomatic, as described in the literature [18,19]. Early initiation of systemic therapy is pivotal for a good prognosis. Patients with suspected infectious disease should always be referred to ophthalmologic evaluation as part of the diagnostic framework, as neuroretinitis can be the first manifestation of miliary TB [20].

### 3.2. Pediatric Ocular Tuberculosis: What Is Known

Pediatric ocular TB is a rare disease. An extensive literature consisting mainly of case reports, small case series, and narrative reviews is available. Phlyctenular keratoconjunctivitis, orbital disease, dacryoadenitis, retinal vasculitis, serpiginous-like choroiditis, multifocal choroiditis, neuroretinitis, and optic nerve involvement have been described. Given the immaturity of children’s immune systems, especially in the youngest, manifestations can be dramatic. This is why most of the published reports describe cases of severe ocular TB [13,14,15,17,20], emphasizing the intensity of ocular inflammation and the gravity of the prognosis. On the other hand, the present case highlights the importance of a prompt diagnosis with only early signs of disease. In this sense, it is interesting that despite the presence of papilledema, chorioretinitis, and central involvement, there were no ophthalmic symptoms.

In everyday practice, mild cases might be overlooked. Our patient presented with mild optic nerve head swelling on fundus ophthalmoscopy and small patches of multifocal chorioretinitis (Figure 1). Papilledema is a sign of intracranial hypertension. In miliary tuberculosis, it may be due to tuberculous meningitis, obstructive hydrocephalus, cerebral edema, or space-occupying lesions such as tuberculomas. In children, papilledema can be mild and associated with preserved visual acuity, thus not excluding the presence of central disease [18,19]. In our case, the detection of small patches of chorioretinitis led to the hypothesis of miliary tuberculosis. Once again, this supports the importance of ophthalmic evaluation as a diagnostic tool in the pediatric workup.

### 3.3. Ultrasonography and MRI: The Importance of Neuroimaging

In our case, the increased optic nerve sheath diameter on ocular ultrasound supported the suspicion of papilledema, helping to exclude optic disc drusen as a differential diagnosis. In children, ocular ultrasonography is especially useful since it is rapid, noninvasive, repeatable, and well tolerated.

The performance of brain MRI is mandatory in all cases of optic nerve involvement, even in grade 1 disc edema, especially in a febrile child. The main brain caseous lesion, along with the intra-axial lesions found at MRI, was pathognomonic for TB. Because of its potentially life-threatening underlying causes, as extensively recommended in the literature, optic disc edema requires a prompt and multidisciplinary evaluation in order to identify its etiology. This is particularly true when associated with systemic or neurological symptoms, such as fever, vomiting, irritability, or hyponatremia [21,22,23]. Neuroimaging was repeated throughout the follow-up period in order to assess the regression of brain lesions. ATT was stopped once residual gliosis in the absence of enhancement was detected. This is consistent with the literature, as radiological resolution or stabilization is considered a valid endpoint for discontinuing antitubercular therapy [24].

### 3.4. Antituberculosis Therapy

Treatment for ocular TB consists of the association of four drugs (isoniazid, rifampicin, pyrazinamide, and ethambutol) for at least two months. Given the poor cerebrospinal fluid penetration of ethambutol and the extensive CNS involvement, a quinolone with high bioavailability and proven efficacy in pediatric TB was added to optimize ATT [15]. Steroids are usually recommended alongside ATT for the treatment of CNS tuberculosis to reduce cerebral edema and vasculitis, limit flare-ups of latent disease, and control paradoxical manifestations associated with an immune reconstitution inflammatory syndrome, as a consequence of ATT onset in severe and disseminated cases [15,16,25,26]. In our case, the initial lack of response to oral ATT raised suspicion of poor intestinal absorption, paradoxical reaction, or concomitant infections or complications. However, when the diagnosis of miliary TB was confirmed, the addition of dexamethasone and the switch to intravenous treatment led to optimal disease control. Interestingly, the child’s irritability—initially mistaken for a personality trait typical of an only child—completely resolved with treatment, confirming its neurological origin. A close follow-up is mandatory for patients undergoing long-term ATT to detect early side effects, including toxic optic neuropathy, as well as long-term efficacy and the chance of relapse [16].

### 3.5. What Is New?

To our knowledge, this is the first article to describe a case of ocular tuberculosis without any ophthalmological symptoms. Even though ocular TB has been described in patients with no systemic symptoms [27,28], only cases of paucisymptomatic ocular tuberculosis have been reported [29]. It is commonly believed that ocular or central involvement should be associated with marked neuro-ophthalmic symptoms. Our patient, instead, presented with preserved visual acuity, no pain, and no red eye. This strengthens the assumption that ophthalmologists should always be involved in the diagnostic workup of infective and immunologic disease, even when no visual impairment has been reported. This is especially true in pediatric routine care, as children’s complaints might be unreliable. In our experience, a multidisciplinary approach, involving infectious disease specialists, pediatricians, ophthalmologists, and neuroradiologists, granted a prompt diagnosis, the escalation of antituberculosis therapy, and the complete resolution of the disease. Moreover, it is remarkable that only a few case series provide such a long follow-up [30,31]: at the five-year follow-up, the child presented good clinical conditions and showed age-appropriate neurodevelopment, with no relapses recorded.

## 4. Conclusions

Ocular tuberculosis still represents a diagnostic challenge, particularly in non-endemic countries and in children. The presented case report shows that pediatric ocular tuberculosis can be clinically silent, even when associated with extensive central involvement. Asymptomatic papilledema with preserved visual acuity should not be overlooked, especially in the presence of systemic symptoms such as unexplained fever or irritability. Fundus examination, supported by ocular ultrasonography and neuroimaging, can reveal an otherwise occult miliary dissemination. A prompt diagnosis allows early initiation of therapy and a better prognosis. A multidisciplinary approach, involving infectious disease specialists, pediatricians, ophthalmologists, and neuroradiologists, should be adopted. Although ocular tuberculosis is rare in non-endemic countries, all ophthalmologists should consider it in the differential diagnosis of neuroretinitis, especially in pediatric and immunocompromised patients.

## Data Availability

The original contributions presented in this study are included in the article. Further inquiries can be directed to the corresponding author.

## Abbreviations

The following abbreviations are used in this manuscript:

TB	Tuberculosis
ATT	Antituberculosis therapy
MTB	Mycobacterium Tuberculosis
TST	Tuberculin skin testing
IGRA	Interferon gamma release assays
PCR	Polymerase chain reaction
EBV	Epstein-Barr Virus
CT	Computed Tomography
CMV	Citomegalovirus
HIV	Human Immunodeficiency Virus
BCVA	Best Corrected Visual Acuity
MRI	Magnetic Resonance Imaging
EEG	Electroencephalogram
CNS	Central Nervous System
CSF	cerebrospinal fluid
IIH	idiopathic intracranial hypertension
MRV	magnetic resonance venography
TBM	tuberculous meningitis
